# Integrating Cognitive Factors and Eye Movement Data in Reading Predictive Models for Children with Dyslexia and ADHD-I

**DOI:** 10.16910/jemr.16.4.6

**Published:** 2024-03-21

**Authors:** Norberto Pereira, Maria Armanda Costa, Manuela Guerreiro

**Affiliations:** NeuroCog – Centro Reabilitação Lesão Cerebral, Centro Linguística Universidade Lisboa (CLUL), Portugal; RISE-HEALTH – Polo de Gestão da Escola Superior de Saúde do Instituto Politécnico do Porto, Portugal; Escola Superior de Saúde Dr. Lopes Dias (ESALD), Portugal; www.neurocog.pt; CLUL, University of Lisbon, Portugal; IMM, Faculty of Medicine, University of Lisbon, Portugal

**Keywords:** dyslexia, ADHD-I, eye-tracking, eye movement, predictive reading models

## Abstract

This study reports on several specific neurocognitive processes and eye-tracking predictors of reading outcomes for a sample of children with Developmental Dyslexia (DD) and At-tention-Deficit/Hyperactivity Disorder – inattentive subtype (ADHD-I) compared to typical readers. Participants included 19 typical readers, 21 children diagnosed with ADHD-I and 19 children with DD. All participants were attending 4th grade and had a mean age of 9.08 years. The psycholinguistic profile of each group was assessed using a battery of neuropsy-chological and linguistic tests. Participants were submitted to a silent reading task with lex-ical manipulation of the text. Multinomial logistic regression was conducted to evaluate the predictive capability of developing dyslexia or ADHD-I based on the following measures: (a) a linguistic model that included measures of phonological awareness, rapid naming, and reading fluency and accuracy; (b) a cognitive neuropsychological model that included measures of memory, attention, visual processes, and cognitive or intellectual functioning, and (c) an additive model of lexical word properties with manipulation of word-frequency and word-length effects through eye-tracking. The additive model in conjunction with the neuropsychological model classification improved the prediction of who develops dyslexia or ADHD-I having as baseline normal readers. Several of the neuropsychological and eye-tracking variables have power to predict the degree of reading outcomes in children with learning disabilities.

## Introduction

Developmental dyslexia (DD) and attention-defi- cit/hyperactivity disorder (ADHD) are two of the most common neurodevelopmental disorders, and each of them occurs in approximately 5% of the population (3, 4). These disorders co-occur more frequently than expected by chance in both population and clinical-based samples (25%–40% of individuals with ADHD meet criteria for DD, 15%–40% of individuals with DD meet criteria for ADHD, and the comorbidity rate between ADHD and learning disabilities is 45.1% (20; 99), suggesting that this comorbidity is not a consequence of selection bias.

According to Smyrnakis et al. (75), cognitive skills, such as visual scanning, selective focusing, retrieving information from lexical storage and short-term memory are essential for reading and can have a direct impact on the individual’s life. Traditionally, neuropsychological models of neurodevelopmental disorders have proposed that a single primary neurocognitive deficit was sufficient to explain all of the symptoms observed for a disorder (e.g., 12; 68). However, findings from several studies have challenged the validity of the single cognitive deficit model (for a review see, 29). In the attempt to explain the cause of comorbidity and the presence of a considerable overlap of neurocognitive deficits between neurodevelopmental disorders, some researchers have suggested a multiple cognitive deficit model for understanding “complex” neurodevelopmental disorders (48; 61; 62; 97).

According to several authors, two specific language processes have been consistently and strongly demonstrated to be a key skill set underlying the successful development of reading skill (24; 40; 67). These processes are phonological awareness (42; 41; 87; 91, 92), and RAN (14; 100). Deficits in phonological awareness and naming speed have been demonstrated to be characteristic of individuals with developmental dyslexia and those who struggle to acquire basic reading skills. Past research has shown that phonological awareness and RAN are distinct constructs but related in their prediction of reading processes (24). The two skills may also have a different developmental course, with phonological awareness applying more influence on early, sub-lexical decoding-dependent tasks and RAN exerting more influence on later, word identification and fluency-dependent tasks related to the lexical route of reading (28; 60; 101). These two skills yielded the strongest effect sizes, substantially higher than effects for behaviour, orthography, memory, and IQ (1; 24; 57). Other factors have also been associated with reading, namely several specific neurocognitive processes such as some measure of IQ and memory measures (1; 57). Some studies have tested IQ as a moderator effect but have not found evidence of differential growth or change along these dimensions (25; 43; 51). Several cognitive and neuropsychological constructs have demonstrated empirical evidence of association with reading processes. Nevertheless, many of them have not been studied as predictors of responsiveness simply because of the focus on the reading-related language processing factors studied most to date, phonological awareness and RAN (24; 40). The process of encoding or representing linguistic information for later analysis and synthesis is a cognitive skill that underlies phonological awareness, grapheme, and the development of individual word identification. These processes have been studied at several levels of resolution, including the individual phoneme and the morpheme (24). There is strong evidence that these processes underlie vocabulary development (27; 50) and spoken language comprehension (10). Scarborough (71) showed that verbal memory substantially predicted reading achievement in school aged children, but only for normal developing readers. Furthermore, Gathercole et al. (26) reported that phonological memory at the phoneme and word levels was a significant predictor of reading for atypical readers, though weaker in predictive power than complex working memory tasks. One form of assessing phonological coding is through pseudoword repetition whose relationship with reading has been systematically reviewed by Gathercole et al. (26). The key dynamic in this relationship is that the ability to repeat pseudowords is an index of the overall quality of the phonological storage system, involved in vocabulary, word learning, and phonological awareness.

A review comparing children with and without developmental dyslexia on measures of working memory and short-term memory concluded that phonological memory measured in tasks such as pseudoword repetition was significantly impaired for atypical readers (82). These authors also found that – in the fully partial model that controlled for the influence of working memory and attention – only phonological memory among measures of short-term memory was retained as significantly impaired in the group with developmental dyslexia. Across several studies and meta-analyses, no systematic differences in the reading achievement of atypical readers that are attributable to IQ have been shown to exist (32; 33; 73, 74; 78). Most previous studies have examined global intelligence for potential direct relationships to reading achievement. The past two decades have witnessed the emergence of more investigations regarding the role of intelligence. Tiu et al. (85) tested a structural model of reading across normal readers and children with developmental dyslexia and found that performance IQ was related to reading comprehension, but only for atypical readers and only as mediated by decoding skill. Consistent with these results, other authors showed that IQ moderated the relationship between reading outcome and specific phonological deficits, such that high IQ poor readers manifested more severe phonological reading deficits (36). Another way in which the IQ–reading relationship has been obscured is that many studies have not considered the well-defined factors that constitute global intelligence scores (24). Vellutino et al. (90) reported correlations between reading achievement and verbal and performance IQ factors at several points from Grade 1 through Grade 4. According to Frijters et al. (24), there is enough research and knowledge about the development of reading processes to suggest that short-term memory, visual memory, and IQ are important factors. According to the latter authors, little research is available to suggest whether any of these factors moderate degree of response to reading intervention among struggling readers.

Neurocognitive deficits in atypical readers encompasses problems with accurate or fluent word recognition, poor decoding, and poor spelling abilities (53). These traits typically result from a phonological deficit and are not better accounted for by intellectual disabilities, sensory impairments, or inadequate educational instruction (4; 44). Deficits in phonological awareness and RAN relative to chronological-age-matched controls and/or reading-level-matched controls have been consistently found in children with developmental dyslexia in transparent (86), intermediate (13; 52), and opaque orthographies ( 16; 40). Phonological awareness is the most relevant predictor of reading decoding in children with developmental dyslexia and normal readers, whereas RAN is more related to reading fluency (102). Although many studies have consistently found that the phonological domain is the most relevant endophenotype of developmental dyslexia (23; 67; 89), atypical readers also have weaknesses in several other neurocognitive domains. For example, children with developmental dyslexia had significant difficulties in the phonological loop and the central executive components (56; 82) of Baddeley’s Working Memory model (9). Mixed results were found in the visuospatial sketchpad component (53). Although most studies have not shown visuospatial short-term memory deficits in individuals with developmental dyslexia (6; 39), others have suggested the presence of significant differences (49). Moreover, working memory plays an important role in the development of reading skills. Specifically, the phonological loop and the central executive components predicted variance in reading decoding, reading fluency, and reading comprehension (56; 58; 82; 81) even after controlling for other neurocognitive variables that are known to be strong predictors of reading, namely phonological awareness and RAN (13; 102). Almost all studies investigating phonological loop capacity have documented reductions in verbal span in children with developmental dyslexia (39; 49; 82; 99). Nonetheless, the literature has been discordant concerning which phonological loop subcomponents are compromised (56). Some researchers have observed that the deficit appeared to be specific to the store mechanism (a reduced phonological similarity effect, i.e., rhyming items are more difficult to remember than nonrhyming items), while the subvocal rehearsal mechanism remained intact. However, others have found that children with developmental dyslexia exhibited less-efficient rehearsal processes (a reduced word-length effect, i.e., short words are easier to remember than sequences of long words) or that phonological similarity and word-length effects did not differ between atypical and typical readers (38; 66; 77). Moreover, some researchers have found an association between phonological loop and articulatory/speech rate (i.e., the number of verbal items repeated per second), suggesting that children with developmental dyslexia experience phonological loop impairments due to their slow articulation rates, which cause phonological loop to function less efficiently (38; 47). The phonological loop also plays an important role in the development of reading skills. A large number of studies have demonstrated that the phonological loop predicts reading decoding (34; 38; 64) and reading comprehension (30; 80). Other researchers have found that the phonological loop did not uniquely predict reading after controlling for phonological awareness and naming speed tasks (60). In comparison to typical readers, atypical readers revealed difficulties in a range of other specific executive functions that include shifting (45), processing speed (72), inhibition (99), and verbal fluency (88). Group differences on several of these executive functions tasks remained significant after general intellectual ability was statistically controlled (54; 99). Taken together, these findings from the literature provide evidence of the multiple cognitive deficit hypothesis.

In the context of ADHD, a substantial body of research consistently shows that children diagnosed with ADHD performed poorly on measures of processing speed (72; 99), inhibition (12), working memory (2), verbal fluency (83), and set shifting ( 70). Willcutt et al. (99), conducted a meta-analytic review of 83 studies and found that groups with ADHD exhibited significant impairments on all executive functioning tasks, on measures of response inhibition, vigilance, working memory, and planning. Significant weaknesses were observed in executive functions tasks among both clinic-referred and community samples, and these weaknesses could not be accounted for by differences in intelligence, academic achievement, or symptoms of other disorders (53). Similarly, in a meta-analytic review conducted by Kasper et al. (37), examining 45 studies on working memory performance in children with ADHD, statistically significant differences were observed with large effect sizes when compared to typical readers in both verbal and visuospatial short-term memory measures. In addition to the well-documented relation between executive functions and ADHD symptoms, other studies have suggested that children with ADHD also exhibit weakness in other neurocognitive measures, which is consistent with the multiple cognitive deficit hypothesis (53). Although various studies did not find phonological processing deficits in children with ADHD (31; 98), others have demonstrated that phonological awareness and RAN deficits are not limited to developmental dyslexia and are also observed in children with ADHD (19; 96). Children with ADHD are also slower or less accurate than typically developing children on measures of complex sentence comprehension (93), lexical and/or sublexical route processing (19; 99), textual organization, and spelling and punctuation errors (46).

Through an in-depth analysis of the interplay between specific neurocognitive processes and eye-tracking metrics involved in reading, our study aims to pinpoint the most influential factors that predict the development of either developmental dyslexia or ADHD-I (inattentive subtype). This exploration of associations offers the potential to inform the design of future focused interventions and provides valuable insights into the underlying mechanisms of developmental dyslexia and ADHD-I. This will be achieved through the application of linear and multinomial logistic regression analysis.

## Methods

### Participants

The participants were 59 Portuguese children, all aged 9 years old (9.08±0.68), with 61% being female. They were native speakers of European Portuguese (L1) and attending the 4th grade. The sample was divided into three distinct neuropsycholinguistic profiles, as follows:

1) Control group: this group comprised 19 individuals, of which 78.9% were female.
2) Developmental Dyslexia: there were 19 children in this group, with 57.9% being female.
3) ADHD-I children: this group included 21 children, with 47.6% being female.

### Criteria for inclusion and procedures

**Control group.** Only children who met the following criteria were included: 1) Portuguese as first language; 2) Wechsler Intelligence Scale for Children-Third Edition (WISC–III) Full Scale IQ ≥ 85 (94, 95); 3) absence of known neurological diseases; 4) absence of sensory (auditory or visual) or motor deficits; 5) exposure to adequate schooling; 6) medium-low minimum socioeconomical level, and 7) average or above average word reading skills assessed on a standardized test of reading fluency and accuracy.

**Developmental Dyslexia.** Inclusion criteria included 1-6 criteria mentioned above plus a) experienced persistent problems in learning to read according to an independent assessment completed by the classroom teacher and, b) reading performance in the lower 15^th^ percentile of the full cohort on a standardized test of reading fluency and accuracy, *O Rei* (17; 18). The diagnosis of dyslexia was discrepancy-based – reading achievement substantially below that expected for age, schooling, and level of intelligence – in accordance with the diagnostic criteria specified in the DSM-IV-R (3).

**ADHD-I**. Children medicated with methylphenidate were excluded from the study. The assessment of ADHD-I (inattentive subtype) was performed according to DSM-IV-R (3) diagnostic criteria.

The neuropsycholinguistic evaluations for the three groups were carried out in schools situated in Lisbon, Portugal. To capture eye movement data, the eye tracking records were conducted at *Centro Linguística Universidade Lisboa (CLUL)*. To ensure ethical considerations, written informed consent was obtained from the next of kin, caretakers, or guardians of the participating children. The study protocol received approval from the Regional Ethical Review Board of the Faculty of Medicine, University of Lisbon, in 2016. Table 1 presents group means for age and IQ, while Table 2 shows demographic characteristic according to gender.

### Psychometric and linguistic measures

#### Intellectual ability

The Portuguese version of the WISC–III (95) was administered to measure general intellectual ability. Our assessment included the utilization of measures such as the Full-Scale IQ, Verbal IQ, and Performance IQ, as well as all the WISC-III subtests.

#### Phonological awareness

ALEPE – *Avaliação da Leitura em Português Europeu* (79) is a comprehensive assessment tool specifically designed to evaluate reading skills in European Portuguese. The battery encompasses various subtests that assess different aspects of reading, including phonological awareness, rapid naming, letter knowledge, word reading, and pseudoword reading. The primary objectives of ALEPE are twofold. Firstly, it aims to determine the child's reading level, taking into consideration their chronological age and educational background. Secondly, it seeks to provide a detailed analysis of the cognitive processes involved in reading. The following tests were selected for assessment purposes: 1) phonemic awareness; 2) rime phonological awareness); 3) uppercase letter reading; 4) word reading - List B; pseudoword reading - List B'; 5) word reading - List C. The phonemic and rime phonological awareness tests assess the individual's ability to manipulate and identify specific phonemes and rhyme patterns in words. The uppercase letter reading test evaluates the individual's proficiency in recognizing and reading uppercase letters. The word reading tests (List B and List C) measure the individual's ability to read real words, while the pseudoword reading test (List B') assesses their capacity to read made-up words.

#### Reading Comprehension

TCL-3 – *Teste de Compreensão da Leitura* (15) allows for the assessment of reading comprehension skills in children attending the 3^rd^ year of the 1^st^ Cycle of Basic Education. This instrument measures literal comprehension (CL), inferential comprehension (CI), critical comprehension (CC), and reorganization of information (RI). At the first level, CL, the reader is required to extract explicit information from the text. At the second level, CI, the reader is expected to use explicit and implicit ideas and information from the text, as well as their intuition, prior knowledge, and personal experiences to formulate conjectures and hypotheses. The third level, RI, involves analysing, synthesizing, and/or organizing information conveyed in the text. Finally, the fourth level, CC, entails the formulation of personal judgments, distinguishing between reality and fantasy, fact, and opinion, evaluating the author's style, characterizing the characters, detecting, and evaluating the author's points of view, among other reactions to perceived messages and the aesthetic qualities of a work.

#### Reading fluency and accuracy assessment

*O Rei* – *Teste de Avaliação da Fluência e Precisão da Leitura* (17; 18) is an assessment instrument designed to evaluate the accuracy and fluency of reading in children from 2^nd^ to 6^th^ grade. Its purpose is to measure a child's performance in reading aloud. Smyrnakis et al. (75) found that in the context of this task, it is necessary to synchronize the pronunciation of phonemes with the continuous visual scanning of the text. This test was administered individually, and it is a simple and quick assessment, allowing for the characterization of a child's performance compared to their peers in terms of both grade level and chronological age. According to the authors, this test demonstrates good psychometric properties in terms of reliability and validity. The selected dependent variables to assess levels of fluency were speed (number of correct words read per minute) and accuracy (percentage of errors), measured after 1 and 3 minutes of reading.

**Table 1.** Group mean for age and IQ.

Measures	Control		Dyslexia		ADHD-I	
	n*_i_* = 19		n*_i_* = 21		n*_i_* = 19	
	Mean	*SD*	Mean	*SD*	Mean	*SD*
Age	9.26	0.15	8.95	0.12	9.05	0.18
Verbal IQ	102.8	3.7	98.9	3.0	83.5	2.9
Performance IQ	103.0	4.5	98.2	2.4	80.9	1.9
Full-Scale IQ	102.5	4.0	97.5	2.5	78.5	1.9

Note. *SD* = Standard deviation.

**Table 2.** Demographic characteristic of the sample.

Sex					
Control n*_i_*		Dyslexia n*_i_*		ADHD-I n*_i_*	
Male	Female	Male	Female	Male	Female
4	15	8	11	11	10

### Eye-Tracking measures

To analyse each target word as a region of interest, the following dependent variables were selected: 1) fixation count (FC); 2) single fixation duration (SFD); 3) first pass reading time (FPRT); 4) second pass reading time (SPRT) and 5) total fixation time (TFT). The latter measure corresponds to the sum of the FPRT with the SPRT. In data analysis, to answer the hypotheses formulated, Frequency (2 levels: low and medium frequency) x Length (3 levels: short, medium, and long length words) interaction effects on eye tracking variables were measured through durations and frequencies of fixations that landed on the target words, as also with FPRT and SPRT.

Pereira et al. (63) provide more comprehensive information regarding the eye-tracking stimuli used in this study.

### Materials

Ocular eye movements were recorded with SMI IVIEW X^TM^ HI-SPEED eye tracking system (SensoMotoric Instruments) (84). This video-based eye tracking compares the relative position of the pupil with the reflex coming from the cornea to calculate the ocular position at a sampling rate of 1250 Hz. This equipment was used to track eye position over time, sampling the horizontal and vertical position of the dominant eye (monocular). Under well controlled experimental conditions, the system afforded a tracking resolution of 0.01º with a gaze position accuracy of 0.25-0.5º, as per the manufacturer’s specification. Fixations were calibrated using 9-13 dots that randomly appeared in a 17-inch screen. The spatial accuracy of the equipment is 0.5º and to limit participant´s head movement a chin and forehead rest was deployed to minimize head movements and stabilize the viewing distance at 550 mm. The eye movements recording through eye tracking was collected at the Psycholinguistics Laboratory of the Faculty of Letters, University of Lisbon.

Word frequency in Portuguese language was determined through the use of "Multifunctional Lexicon Computing of Contemporary Portuguese"(5) and ESCOLEX (76) databases. For frequency, words were divided in two intervals: 1) low-frequency words (LF) - [0-1000] Token and 2) medium-frequency words (MF) - [1001-10000] Token. Regarding word-length, the criteria related to the size of the perceptual window and word size were the following (we have adjusted the criteria used by Hyönä & Olson,^35^) to Portuguese: 1) short words (S) - [4-6] letters; 2) medium words (M) - [7-10] letters and 3) long words (L) - [11-14] letters (Table 3).

**Table 3.** Word classification according to their frequency and length.

Stimuli		
Length	Short (S)	[4 - 6] Letters
	Medium (M)	[7 - 10] Letters
	Long (L)	[11 - 14] Letters
Frequency	Low (LF)	0 - 1000 Token
	Medium (MF)	1001 - 10000 Token
Length x Frequency	(S + LF)	*Corais* (corals)
	(S + MF)	*Equipa* (team)
	(M + LF)	*Marinhas* (marine)
	(M + MF)	*Conhecer* (to know)
	(L + LF)	*Mergulhadores* (sea divers)
	(L + MF)	*Investigação* (research)

### Procedure

We first determined the neuropsycholinguistic profile of each group. The neuropsychological and linguistic evaluations included instruments to assess intellectual performance, verbal working memory, short-term verbal memory, visual attention, phonological awareness, reading comprehension and reading fluency and accuracy. Following this phase, each group underwent a reading task in which text lexical properties were controlled, and eye movements were recorded.Parte superior do formulário Target words were distributed throughout the text to prevent them from being placed at the end of the paragraph and close to punctuation marks, locus favorable to wrap-up effects that we aimed to avoid, because they can be easily confused with the lexical properties of the words themselves. Also, contiguities between target words were avoided to mitigate spill over and agglomeration effects, that could hinder the analysis of eye movements. For the final on-screen version, prioritizing readability and facilitating subsequent eye movement data analysis, we selected Courier New, a non-proportional font, size 22, and used double line spacing. The text was divided into three parts, each presented on a separate slide on a 17-inch screen. At the end of each slide, the transition to the next slide was initiated through ocular fixation on the top right corner of the screen. The main experiment was preceded by a set of instructions and a pre-test. Calibration of the eye tracker was conducted using 9-13 fixation points that appeared randomly in the visual field where the text was displayed. Monocular recording was performed on the dominant eye, and eye dominance had been determined prior to the start of the experiment. The pre-test involved silent reading of a training text followed by three multiple-choice questions aimed at assessing the level of comprehension. Including a comprehension questionnaire after reading the text ensured that the reader identified the words, accessed their meanings, and integrated them into broader syntactic and discursive structures. After this step, the equipment was recalibrated following the previously described parameters to commence the silent reading of the main text. Upon completing the reading, participants responded to three multiple-choice questions to assess their comprehension level. The questions primarily served to encourage subjects to read for comprehension and to identify participants who couldn't answer at least 2 out of 3 questions. It's important to note that the comprehension outcomes were not utilized at any stage of our analysis. In total, we allocated 180 minutes to each child for data collection, which was divided into three sessions. This included two sessions of 75 minutes each for conducting neuropsycholinguistic assessments and an additional 30 minutes to collect eye movement data.

Further details and a more comprehensive discussion on these formatting choices, stimuli and their impact on reading and eye movement analysis, may be found in Pereira et al. (63).

### Statistical analysis

We assessed ocular movement behaviour in each of the three groups by selecting eye-tracking variables and employed both parametric and non-parametric statistical methods. The normality assumption was confirmed using the *Shapiro-Wilk* normality test. To analyse the data, we conducted multivariate analysis, initially using the *Anova F* statistic to assess equality of variances. When variances were found to be equal, multiple comparisons were carried out using the *Tukey HSD* test. In cases where variances were not equal, the *Brown-Forsythe* statistic was used as an alternative to the *Anova F* statistic, followed by the *post-hoc Games-Howell* test. In instances where the normality assumption was violated, we employed the *Kruskal-Wallis* test for independent samples.

To classify the three groups based on the values ​​of the predictor variables and determine the weight of the dependent variables in each of them, multinomial logistic regression was used. Using a stepwise selection approach, we carefully chose variables to construct a well-balanced model capable of forecasting the outcomes of the dependent variables, dyslexia and ADHD-I. This model relies on the independent variables, encompassing cognitive factors and eye-tracking measures, which constitute the focus of our investigation. The assumptions of the model were analyzed, namely that of normal distribution, homogeneity, and independence of errors. The first two assumptions were graphically validated, and the independence assumption was validated with the *Durbin-Watson* statistic. The VIF was used to diagnose multicollinearity. Outlier observations were also eliminated (e.g., observations with a studentized residue, in absolute value, greater than 1.96). To estimate the weight of independent variables *x* in the expected value of a dependent variable *y*, linear regression was used through the stepwise method. For linear regression, *Gaus-Markov* conditions were verified, namely, residuals with zero mean, constant variance, and normal distribution of the residuals. Throughout our analyses, we considered a Type I error probability (α) of 0.10. This choice allows for a slightly higher alpha level, which can aid in promptly identifying variables or patterns that merit further investigation and reduces the risk of missing important findings.

The assumptions for using the different statistical methods described above were as described in Marôco (2014) and Pestana & Gageiro (2014). Statistical analysis was performed using the IBM SPSS Statistics Version 25.

## Results

### Neurocognitive measures

The comparison of the cognitive performance between the three groups (Table 4), suggest that children with ADHD-I show lower performance across various cognitive domains, including verbal and performance IQ, verbal comprehension, perceptual organization, processing speed, working memory, attention span, vocabulary, information processing, verbal reasoning, and executive functioning. These results highlight the cognitive differences between individuals with ADHD-I and those with developmental dyslexia or typically developing individuals. Atypical readers showed specific difficulties in subtests that require the phonological loop of Baddeley’s (7, 8, 9; 10; 11) multicomponent model of working memory compared to typically developing children. However, they performed better than the ADHD-I group in perceptual organization, vocabulary, information processing, and verbal reasoning.

**Table 4.** Means, standard deviations, medians, 1^st^ and 3^rd^ percentiles, ANOVA and *Kruskal-Wallis* independent samples: WISC-III composite and subtest results.

Measures	Groups	Mean (*SD*)	$\tilde{X}$(*Q3-Q1*)	Multiple Comparisons
Verbal IQ	Control	102.85 (13.20)		Control ≠ ADHD-I (*p* = 0.000)^1.2^ Dyslexia ≠ ADHD-I (*p* = 0.002)^1.2^
	Dyslexia	98.89 (13.14)		
	ADHD-I	83.45 (13.02)		
Performance IQ	Control		106.00 (115.50 – 87.00)	ADHD-I ≠ Dyslexia (*p* = 0.000)^5^ ADHD-I ≠ Control (*p* = 0.000)^5^
	Dyslexia		95.00 (105.00 – 90.00)	
	ADHD-I		79.50 (89.00 – 73.00)	
Full IQ	Control		98.00 (117.00 – 89.50)	ADHD-I ≠ Dyslexia (*p* = 0.000)^5^ ADHD-I ≠ Control (*p* = 0.000)^5^
	Dyslexia		98.00 (105.00 – 88.00)	
	ADHD-I		78.00 (84.75 – 74.25)	
Verbal Comprehension Index (VCI)	Control	104.08 (13.43)		Control ≠ ADHD-I (*p* = 0.000)^1.2^ Dyslexia ≠ ADHD-I (*p* = 0.001)^1.2^
	Dyslexia	100.58 (11.72)		
	ADHD-I	84.50 (13.93)		
Perceptual Organization Index (VSI)	Control		106.00 (118.00 – 91.50)	ADHD-I ≠ Dyslexia (*p* = 0.009)^5^ ADHD-I ≠ Control (*p* = 0.001)^5^
	Dyslexia		98.00 (103.00 – 86.00)	
	ADHD-I		87.00 (91.00 – 72.00)	
Processing Speed Index (PSI)	Control	102.31 (21.67)		Dyslexia ≠ ADHD-I (*p* = 0.002)^3.4^
	Dyslexia	102.68 (13.78)		
	ADHD-I	87.37 (10.89)		
Digit Span Total	Control	10.62 (2.43)		Control ≠ Dyslexia (*p* = 0.023)^1,2^ Control ≠ ADHD-I (*p* = 0.027)^1,2^
	Dyslexia	8.42 (2.06)		
	ADHD-I	7.40 (2.26)		
Forward Digit Span	Control	7.62 (1.12)		Control ≠ Dyslexia (*p* = 0.041)^1,2^ Control ≠ ADHD-I (*p* = 0.008)^1,2^
	Dyslexia	6.47 (1.31)		
	ADHD-I	6.20 (1.32)		
Backwards Digit Span	Control		5.00 (5.00 – 4.00)	ADHD-I ≠ Control (*p* = 0.042)^3^
	Dyslexia		3.00 (5.00 – 3.00)	
	ADHD-I		3.00 (4.00 – 3.00)	
Vocabulary	Control		10.00 (14.00 – 9.00)	ADHD-I ≠ Dyslexia (*p* = 0.000)^5^ ADHD-I ≠ Control (*p* = 0.000)^5^
	Dyslexia		10.00 (12.00 – 9.00)	
	ADHD-I		6.00 (8.00 – 5.00)	
Information	Control		9.00 (11.00 – 7.00)	ADHD-I ≠ Control (*p* = 0.036)^5^
	Dyslexia		8.00 (11.00 – 6.00)	
	ADHD-I		6.00 (9.00 – 6.00)	
Similarities	Control		13.00 (14.00 – 9.50)	ADHD-I ≠ Dyslexia (*p* = 0.036)^5^
	Dyslexia		12.00 (13.00 – 11.00)	
	ADHD-I		9.00 (11.50 – 8.00)	
Comprehension	Control	10.46 (2.47)		n.s.
	Dyslexia	9.94 (1.95)		
	ADHD-I	7.39 (2.45)		
Block design	Control	10.92 (2.53)		Control ≠ ADHD-I (*p* = 0.000)^1,2^ Dyslexia ≠ ADHD-I (*p* = 0.000)^1,2^
	Dyslexia	9.59 (2.15)		
	ADHD-I	6.44 (2.75)		
Object assembly	Control		9.00 (11.50 – 7.50)	ADHD-I ≠ Control (*p* = 0.041)^5^ ADHD-I ≠ Dyslexia (*p* = 0.006)^5^
	Dyslexia		9.00 (10.00 – 8.00)	
	ADHD-I		7.50 (9.00 – 4.25)	
Pictures completion	Control	11. 08 (3.20)		Control ≠ ADHD-I (*p* = 0.012) ^1,2^
	Dyslexia	10. 24 (2.73)		
	ADHD-I	8.56 (2.41)		
Picture arrangement	Control	9.92 (3.30)		n.s.
	Dyslexia	10.47 (3.20)		
	ADHD-I	8.72 (2.08)		
Coding	Control	11.08 (3.88)		Control ≠ ADHD-I (*p* = 0.019)^3,4^ Dyslexia ≠ ADHD-I (*p* = 0.015)^3,4^
	Dyslexia	9.82 (2.63)		
	ADHD-I	7.39 (2.25)		
Symbol search	Control	9.77 (4.38)		Dyslexia ≠ ADHD-I (*p* = 0.003)^3,4^
	Dyslexia	11.53 (2.94)		
	ADHD-I	7.89 (2.14)		
Arithmetic	Control	9.85 (3.11)		n.s.
	Dyslexia	9.18 (2.83)		
	ADHD-I	8.17 (1.98)		
Mazes	Control	11.31 (2.98)		n.s.
	Dyslexia	11.56 (2.28)		
	ADHD-I	9.68 (3.16)		

Note: ^1^*ANOVA F* Test; ^2^*Tukey HSD*; ^3^*Brown-Forsythe* statistic; ^4^*Games-Howell*;^5^*Kruskal-Wallis* independent samples; *SD* – Standard deviation; $\tilde{X}$ – Median, *Q3* – 3^rd^ percentile, *Q1* – 1^st^ percentile.

### Linguistic measures

Regarding phonemic awareness (Table 5), the study examined various measures related to the phonological structure of words, specifically consonant-vowel (CV) syllables, non-common consonant-vowel (nCV) syllables, consonant-vowel-consonant (CVC) syllables and non-common consonant-vowel-consonant (nCVC) syllables in the three groups studied. Overall, there were no statistically significant differences observed among the groups on phoneme discrimination tasks.

**Table 5.** Means and standard deviations. Phonemic awareness: ALEPE.

Measures	Groups	Mean (*SD*)	Multiple Comparisons
CV	Control	5.85 (0.38)	n.s.
	Dyslexia	5.59 (0.62)	
	ADHD-I	5.14 (1.70)	
nCV	Control	4.00 (0.00)	
	Dyslexia	3.94 (0.24)	
	ADHD-I	3.64 (1.08)	
Total_CV	Control	9.85 (0.38)	
	Dyslexia	9.53 (0.62)	
	ADHD-I	8.79 (2.67)	
CVC	Control	5.23 (0.93)	
	Dyslexia	4.94 (1.14)	
	ADHD-I	4.29 (2.20)	
nCVC	Control	4.00 (0.00)	
	Dyslexia	3.88 (0.33)	
	ADHD-I	3.57 (1.09)	
Total_CVC	Control	9.23 (0.93)	
	Dyslexia	8.82 (1.24)	
	ADHD-I	7.86 (2.96)	
Total Sum	Control	19.08 (1.19)	
	Dyslexia	18.35 (1.66)	
	ADHD-I	16.64 (5.24)	

Note: n.s. – not significant; CV – consonant-vowel; nCV – non-common consonant-vowel syllable; CVC – consonant-vowel-consonant syllable; nCVC – non-common consonant-vowel-consonant. *SD* – Standard deviation.

As far as epilinguistic awareness of rhyme is concerned (Table 6), for most of the speech sound processing measures, there were no statistically significant differences observed among the groups. However, there were significant differences between atypical readers and typically developing children for non-common consonant-vowel-consonant (nCVC) phoneme discrimination, a measure of metalinguistic processing.

**Table 6.** Means, standard deviations, and medians. 1^st^ and 3^rd^ percentiles and *Kruskal-Wallis* independent samples. Epilinguistic awareness of rhyme: ALEPE.

Measures	Groups	Mean (*SD*)	$\tilde{X}$ *(Q3–Q1)*	Multiple comparisons
CV	Control	4.62 (1.33)		n.s.
	Dyslexia	4.53 (1.46)		
	ADHD-I	4.07 (1.64)		
nCV	Control	4.00 (0.00)		
	Dyslexia	3.35 (0.86)		
	ADHD-I	3.29 (1.14)		
Total_CV (CV plus nCV)	Control	8.62 (1.33)		
	Dyslexia	7.88 (1.54)		
	ADHD-I	7.36 (2.65)		
CVC	Control	4.85 (1.46)		
	Dyslexia	5.29 (1.31)		
	ADHD-I	4.36 (1.55)		
nCVC	Control		^a^	Dyslexia ≠ Control (*p* = 0.025)^1^
	Dyslexia		4.00 (4.00 – 3.00)	
	ADHD-I		4.00 (4.00 – 3.00)	
Total_CVC (CVC plus nCVC)	Control	8.77 (1.42)		n.s.
	Dyslexia	8.76 (1.56)		
	ADHD-I	7.36 (2.59)		
Total sum	Control	17.38 (2.40)		
	Dyslexia	16.71 (2.59)		
	ADHD-I	13.86 (5.42)		

Note: n.s. – not significant; CV – consonant-vowel syllable; nCV – non-common consonant-vowel syllable; CVC – consonant-vowel-consonant syllable; nCVC – non-common consonant-vowel-consonant syllable. ^1^*Kruskal-Wallis* independent samples; *SD* – Standard deviation; $\tilde{X}$ – Median, *Q3 –* 3^rd^ percentile, *Q1* – 1^st^ percentile; ^a^nCVC it is constant when Groups = Control. It was omitted.

Regarding isolated word reading processing (Table 7), the results indicate that children with dyslexia and ADHD-I demonstrate lower performance in reading accuracy and speed compared to normal readers. Specifically, the atypical readers had lower scores in reading simple and inconsistent words, as well as a lower percentile of consistent words read correctly, while the ADHD-I group had lower scores in reading inconsistent words and a lower percentile of total words read correctly. Despite these differences, no significant differences were observed among the groups for reaction time measures.

**Table 7.** Means, standard deviations, median, 1^st^ and 3^rd^ percentiles and, *Kruskal-Wallis* independent samples. Written language processing | Word reading: ALEPE.

Measures	Groups	Mean (*SD*)	$\tilde{X}$ *(Q3–Q1)*	Multiple comparisons
Percentage of simple words read correctly	Control	96.15 (5.23)		n.s.
	Dyslexia	88.64 (17.09)		
	ADHD-I	85.83 (15.86)		
Percentage of inconsistent words read correctly	Control	78.85 (16.88)		
	Dyslexia	65.91 (24.85)		
	ADHD-I	60.83 (10.43)		
Percentile of consistent words read correctly	Control		99,00 (99.00 – 99.00)	Dyslexia ≠ Control (*p* = 0.049)^1^
	Dyslexia		25,00 (99.00 – 10.00)	
	ADHD-I		25,00 (99.00 – 10.00)	
Percentile of total words read correctly (Sum of simple, consistent and inconsistent words)	Control		60,00 (92.50 – 25.00)	ADHD-I ≠ Control (*p* = 0.023)^1^
	Dyslexia		25,00 (60.00 – 5.00)	
	ADHD-I		17,50 (40.00 – 4.00)	
Mean reaction time/ms - simple words	Control	1237.69 (253.29)		n.s.
	Dyslexia	1350.27 (455.25)		
	ADHD-I	1236.70 (499.29)		
Mean reaction time/ms - consistent words	Control	1267.00 (351.88)		
	Dyslexia	1382.64 (520.46)		
	ADHD-I	1223.00 (448.92)		
Mean reaction time/ms - inconsistent words	Control	1629.08 (665.52)		
	Dyslexia	1857.09 (1117.02)		
	ADHD-I	1741.10 (765.33)		
Sum of mean reaction times/ms	Control	1377.85 (403.77)		
	Dyslexia	1530.00 (679.91)		
	ADHD-I	1400.20 (533.97)		

Note: n.s. – not significative; ^1^*Kruskal-Wallis* independent sample test; *SD* – Standard deviation; $\tilde{X}$-Median, *Q3*- 3^rd^ percentile, *Q1*-1^st^ percentile.

Considering the performance of the three groups in various measures related to pseudoword reading and reaction times (Table 8), while the control group generally demonstrated higher accuracy rates and faster reaction times, the dyslexia and ADHD-I groups faced challenges in these tasks. However, the observed differences were not statistically significant in most cases, except for the significant difference between the ADHD-I and typically developing children in pseudowords reading accuracy.

**Table 8.** Means, standard deviations, medians, 1^st^ and 3^rd^ percentiles, ANOVA and Kruskal-Wallis independent sample test. Written word processing | Pseudowords reading: ALEPE test. Written word processing | Pseudowords reading: ALEPE.

Measures	Groups	Mean (*SD*)	$\tilde{X}$ *(Q3–Q1)*	Multiple comparisons
Percentage of simple pseudowords read correctly	Control	93.59 (9.72)		n.s.
	Dyslexia	80.31 (19.46)		
	ADHD-I	78.32 (24.61)		
Percentage of consistent pseudowords read correctly	Control	84.62 (8.91)		
	Dyslexia	77.27 (20.11)		
	ADHD-I	65.00 (25.40)		
Percentage of total pseudowords read correctly	Control		91.70 (95.80 – 81.25)	ADHD-I ≠ Control (*p* = 0.040)^3^
	Dyslexia		87.50 (91.70 – 66.70)	
	ADHD-I		81.25 (87.50 – 59.40)	
Mean reaction time/ms - simple pseudowords read correctly	Control	1452.77 (244.21)		n.s.
	Dyslexia	1471.00 (700.19)		
	ADHD-I	1054.60 (498.49)		
Mean reaction time/ms - consistent pseudowords read correctly	Control	1418.00 (392.18)		
	Dyslexia	1527.18 (822.41)		
	ADHD-I	1321.70 (807.24)		
Mean reaction time - total pseudowords read correctly	Control	1431.46 (310.27)		
	Dyslexia	1499.36 (755.42)		
	ADHD-I	2988.40 (5996.48)		
Mean reaction time – Percentile of simple pseudowords read correctly	Control	26.62 (17.12)		Control ≠ ADHD-I (*p* = 0.021)^1,2^
	Dyslexia	36.00 (32.13)		
	ADHD-I	63.90 (34.59)		

Note: n.s. – not significative; ^1^*Brown-Forsythe* statistic; ^2^*Games-Howell*;^3^*Kruskal-Wallis* independent sample test; *SD* – Standard deviation, $\tilde{X}$-Median, *Q3* – 3^rd^ percentile, *Q1*-1^st^ percentile.

When comparing the groups on letter recognition (Table 9), based on the measures of uppercase letter reading, it appears that typically developing children performed slightly better and had higher percentiles compared to atypical readers and children with ADHD-I. However, it's important to interpret these findings with caution, as the observed differences were not statistically significant.

**Table 9.** Means and standard deviations: written word processing. Letter identification: ALEPE.

Measures	Groups	Mean (*SD*)	Multiple measures
Uppercase letter reading	Control	22.85 (0.38)	n.s.
	Dyslexia	21.64 (2.98)	
	ADHD-I	22.60 (0.52)	
Percentile of uppercase letter reading	Control	85.31 (33.42)	
	Dyslexia	64.18 (48.37)	
	ADHD-I	63.40 (45.96)	

Note: n.s. – not significative; *SD* – Standard deviation.

Regarding reading comprehension (Table 10), typically developing children generally had higher mean scores on the comprehension measures compared to atypical readers and children with ADHD-I. Specifically, significant differences were observed in literal comprehension, with typically developing readers having better performances than atypical readers.

**Table 10.** Means, standard deviations, and independent samples ANOVA. Reading comprehension test: TCL-3.

Measures	Groups	M (*SD*)	Multiple Comparisons
Literal comprehension	Control	6.93 (2.09)	Control ≠ Dyslexia (*p* = 0.026)^1,2^
	Dyslexia	4.73 (2.12)	
	ADHD-I	5.50 (2.31)	
Inferential comprehension	Control	4.71 (1.77)	n.s.
	Dyslexia	4.33 (2.29)	
	ADHD-I	3.38 (1.54)	
Critical comprehension	Control	1.07 (0.62)	
	Dyslexia	1.27 (0.70)	
	ADHD-I	0.81 (0.75)	
Reorganization	Control	2.71 (0.99)	
	Dyslexia	1.60 (1.30)	
	ADHD-I	2.13 (1.20)	
Total result	Control	16.07 (5.08)	Control ≠ ADHD-I (*p* = 0.033)^1,2^
	Dyslexia	11.93 (4.85)	
	ADHD-I	11.50 (4.75)	

Note: n.s. – not significative. ^1^ANOVA F-statistic; ^2^*Tukey HSD*; *SD* – Standard deviation.

Considering reading fluency and accuracy (Table 11), normal readers performed better on all the reading related measures compared to atypical readers and children with ADHD-I. There were statistically significant differences between normal readers and both the atypical readers and children with ADHD-I in terms of correct words read, total reading time, and reading speed. These findings suggest that normal readers exhibited higher reading proficiency, faster reading speed, and more accurate word recognition compared to the atypical readers and children with ADHD-I.

**Table 11.** Medians, 1^st^ and 3^rd^ percentiles and *Kruskal-Wallis* independent samples. Reading Fluency and Accuracy: *O Rei*.

Measures	Groups	$\tilde{X}$(*Q3–Q1*)	Multiple comparisons
Correct words read in 60’’	Control	111.00 (125.00 – 99.50)	Dyslexia ≠ Control (*p* = 0.000)^1^ ADHD-I ≠ Control (*p* = 0.000)^1^
	Dyslexia	71.00 (80.00 – 50.50)	
	ADHD-I	77.00 (85.00 – 46.50)	
Correct words read 180’’	Control	270.00 (274.50 – 265.50)	Dyslexia ≠ Control (*p* = 0.000)^1^ ADHD-I ≠ Control (*p* = 0.001)^1^
	Dyslexia	179.00 (227.50 – 123.50)	
	ADHD-I	177.00 (242.50 – 111.50)	
Total reading time/seconds	Control	153.00 (176.50 – 133.00)	ADHD-I ≠ Control (*p* = 0.001)^1^ Dyslexia ≠ Control (*p* = 0.000)^1^
	Dyslexia	256.00 (381.50 – 211.00)	
	ADHD-I	235.00 (399.50 – 186.50)	
Fluency index - reading speed	Control	90.00 (91.50 – 88.50)	Dyslexia ≠ Control (*p* = 0.000)^1^ ADHD-I ≠ Control (*p* = 0.000)^1^
	Dyslexia	59.67 (75.83 – 41.17)	
	ADHD-I	59.00 (80.83 – 37.17)	

Note: n.s. – not significative. ^1^*Kruskal-Wallis* independent samples; $\tilde{X}$-Median, *Q3*- 3^rd^ percentile, *Q1*-1^st^ percentile.

### Multinomial Logistic Regression

Multinomial logistic regression was used to estimate the probability of children having dyslexia or ADHD-I compared to typically developing children, based on the WISC-III measures of "Backwards Digit Span," "Vocabulary," and "Code," as well as the eye-tracking variable "Fixation counts of low-frequency long length-words." (L+LF_FC). These predictor variables were selected from a larger set of potential predictors that showed no discriminative power. According to the stepwise method, these were the most relevant variables that contribute to the model's predictive power. The adjusted model is statistically significant ( $G^{2}$(8) = 46.774; *p* = 0.000) (see Table 12). The coefficient estimates of the model for the dependent variables and for the classes "children with dyslexia" and "children with ADHD-I" relative to the reference class "typically developing children" are presented in the same table. According to the adjusted model, the transition from the reference class "typically developing children" to the class "children with dyslexia" is not significantly affected by the results obtained in the "Vocabulary" subtest (b_Vocabulary_ = -0.142; *p* = 0.458) or the "Code" subtest (b_Code_ = -0.255; *p* = 0.275). However, the probability of transitioning from the reference class "typically developing children" to the class "children with dyslexia" is significantly affected by the results achieved in the backwards digit span subtest (b_Backwards Digit Span_ = -1.331; *p* = 0.030) and the total number of fixations on long length low-frequency words (b_L+BF_FC_ = 0.107; *p* = 0.018). The odds ratio of transitioning from the "typically developing children" class to the "children with dyslexia" class is 0.264 and 1.113. This means that for every unit increase in "Backwards Digit Span" and "L+BF_FC," the odds of having dyslexia decrease by 73.6% and increase by 11.3%, respectively. Similarly, according to the adjusted model, the transition from the reference class "typically developing children" to the "children with ADHD-I" class is not significantly affected by "Backwards Digit Span" (b_Backwards Digit Span_ = -1.266; *p* = 0.054) or the number of fixations on long length low-frequency words (b_L+BF_FC_ = 0.077; *p* = 0.101). However, the probability of transitioning from the reference class to the "children with ADHD-I" class is significantly affected by the results achieved in the "Vocabulary" (b_Vocabulary_ = -0.702; *p* = 0.008) and "Code" (b_Code_ = -0.794; *p* = 0.016) subtests. The odds ratio of transitioning from the "typically developing children" class to the "children with ADHD-I" class is 0.496 and 0.452. This means that for every unit increase in "Vocabulary" and "Code," the odds of having ADHD-I decrease by 50.4% and 54.8%, respectively. Lastly, this model correctly classifies 81.4% of the cases (see Table 12).

**Table 12.** Adjustment information; Coefficients of the multinomial model that relates children with dyslexia and children with ADHD-I to dependent variables. The reference class is the "typically developing children" class; Model classification.

Model fitting criteria			Coefficients						Classification
Likelihood ratio tests					*B*	Χ²_Wald_	*Sig.*	ÔR	% Correct
Χ²	*df*	*Sig*.	Dyslexia	Intercept	5.558	1.523	0.217		81.4
46.77	8	0.00		Backwards digit span	-1.331	4.700	0.030	0.264	
				Vocabulary	-0.142	0.551	0.458	0.868	
				Coding	-0.255	1.193	0.275	0.775	
				L+LF_FC	0.107	5.641	0.018	1.113	
			ADHD-I	Intercept	16.349	8.513	0.004		
				Backwards digit span	-1.266	3.709	0.054	0.282	
				Vocabulary	-0.702	7.059	0.008	0.496	
				Coding	-0.794	5.763	0.016	0.452	
				L+LF_FC	0.077	2.692	0.101	1.081	

Abbreviations: *df.* – degrees of freedom; Sig. – significance; ÔR – *Odds Ratio*.

Further analysis was conducted using linear regression to determine which variables influence the number of correct words read in 1 and 3 minutes. The goal at this stage was to choose a subset of independent variables for inclusion in the final model. The stepwise method identified the most significant predictors for explaining the 'number of words correctly read in 60 seconds' as follows: 'Diagnostic Recoded Dyslexia' (DiagRecDis), 'Diagnostic Recoded ADHD-I' (DiagRecDA), the WISC-III 'Picture Completion' subtest, and the 'total fixation time of medium-length medium-frequency words' (M+MF_TFT). Multiple linear regression results for the variables were respectively: "DiagRecDA" (*β* = -42.939; *t* (34) = -6.012; *p* = 0.000), "DiagRecDis" (*β* = -43.781; *t* (34) = -6.435; *p* = 0.000), WISC-III Picture Completion subtest (*β* = -2.305; *t* (34) = -2.355; *p* = 0.024) and "M+MF_TFT" (*β* = -0.001; *t* (34) = -2.192; *p* = 0.035).

**Table 13.** Dependent variable: Number of words read correctly in 60 seconds.

Summary model			ANOVA		Dependent variable	Predictors	Non-standardized coefficients	*T*	*Sig.*
							*B*		
$R^{2}$	$R_{a}^{2}$	*Durbin-Watson*	*F*	*Sig.*	Number of correct words read in 60’’.	(Constant)	142.467	11.823	0.000
0.678	0.641	2.475	17.933	0.000		DiagRecDis	-43.781	-6.435	0.000
						DiagRecDA	-42.939	-6.012	0.000
						Picture Completion	-2.305	-2.355	0.024
						M+MF_TFT	-0.001	-2.192	0.035

Abbreviations: Sig. – Significance.

Therefore, our final adjusted model (the formula for calculating the number of correct words read in 60 seconds) is:

Number of correct words read in 60'' = 142.467 - [(42.939 * DiagRecDA) - (43.781 * DiagRecDis) - (2.305 * Picture Completion) - (0.001 * M+MF_TFT)]

This model is significant and explains a high proportion of the variability in the number of words correctly read in 60 seconds (*F* (4, 34) = 17.933; *p* = 0.000; $R_{a}^{2}$ = 0.641) (See Table 13).

Regarding the number of correct words read in 180 seconds, stepwise method identified the most significant predictors for explaining this measure as follows: "DiagRecDA", "DiagRecDis," and “second pass reading time of short length medium-frequency words” (S+MF_SPRT). Multiple linear regression results for the variables were respectively: "DiagRecDA" (*β* = -50.599; *t* (35) = -2.484; *p* = 0.018), "DiagRecDis" (*β* = -66.527; *t* (35) = -3.403; *p* = 0.002), and "S+MF_SPRT" (*β* = -0.015; *t* (35) = -3.013; *p* = 0.005).

**Table 14.** Dependent variable: Number of words read correctly in 180 seconds.

Summary model			ANOVA		Dependent variable	Predictors	Non-standardized Coefficients		*T*	*Sig.*
							*B*			
$R^{2}$	$R_{a}^{2}$	Durbin-Watson		*F*	*Sig.*	Number of correct words read in 180 seconds	(Constant)	273.858	19.951	0.000
0.520	0.479	2.002		12.650	0.000		DiagRecDis	-66.527	-3.403	0.002
							DiagRecDA	-50.599	-2.484	0.018
							S+MF_SPRT	-0.015	-3.013	0.005

Abbreviations: Sig. – Significance.

Our final adjusted model is:

Number of correct words read in 180'' = 273.858 - [(50.599 * DiagRecDA) - (66.527 * DiagRecDis) - (0.015 * S+MF_SPRT)]

This model represents a good fit to the data and explains a proportion of the variability in the number of words read correctly in 180 seconds (*F* (3, 35) = 12.650; *p* = 0.000; $R_{a\;}^{2}$= 0.520) (See Table 14).

## Discussion and Conclusions

The results from the multinomial logistic regression are groundbreaking in that there are not any studies conducted in Portugal or abroad that have focused on predictive models for dyslexia and ADHD-I, incorporating cognitive variables alongside eye movement data during reading tasks. Based on the observations from the multinomial regression model, we can conclude that there are unique cognitive mechanisms underlying the distinct reading difficulties in atypical readers and children with ADHD-I.

Considering Baddeley’s multicomponent model of working memory (2, 8, 9; 10; 11), the phonological loop, often referred as short-term memory, plays an important role in allocating different resources for processing the lexical properties of words in atypical readers. According to Ramus et al. (67), children with dyslexia exhibit significant impairments in phonological skills, which supports the view that their deficits are related to cognitive skills applied to phonological representations. These findings were also found in other studies with children with dyslexia (52; 55; 56). Considering word recognition and morphological processing, atypical readers made more fixations in long length low-frequency words, which means that children with dyslexia activate more visual-attention mechanisms and rely more on phonological representations to decode words, especially when they have low-frequency morphemes. The capacity for phonological recoding stands out, as poor performance in this area acts as a risk factor for developing reading disabilities, which is in agreement with Ramus et al. (67). This finding is also in accordance with the study of Reynolds & Besner (69), who stated that phonological decoding is an attention-demanding process even in skilled adult readers. In particular, graphemic parsing requires an efficient orienting of visual spatial attention (22; 65) in addition to appropriate phonological skills (68; 103). Furthermore, our data do not support Facoetti et al. (21) multisensory deficit of attention hypothesis as a core deficit in developmental dyslexia, which is characterized by poor (i.e., inaccurate) phonological decoding. This lack of support is evident as we did not find a similar pattern in ADHD-I. This suggests that atypical readers may activate attention mechanisms to aid phonological decoding, while the latter group appears not to rely on such mechanisms. Finally, we did not, to our surprise, find significant differences regarding phonemic awareness in almost all measures used. We believe this is due to sample size limitations. However, there were significant differences between atypical readers and typically developing children for non-common consonant-vowel-consonant (nCVC) phoneme discrimination, a measure of metalinguistic processing.

On the other hand, additional risk factors for developing ADHD-I include deficits in lexical memory, difficulties in understanding and using words (knowledge of word meanings), deficits in verbally expressing concepts, as well as impairments in processing speed, short-term visual memory, capacity for automated “mechanical” learning, psychomotor speed, visual perception, ocular-motor coordination, visual scanning ability, cognitive flexibility, visual attention, concentration, and motivation. Unlike atypical readers, individuals with ADHD-I do not appear to activate visual-attention mechanisms and rely less on phonological representations for decoding words. Facoetti et al. (21) multisensory deficit of attention hypothesis appears to provide a better explanation for the reading difficulties observed in children with ADHD-I. In summary, our findings highlight significant intra-individual variability in the cognitive mechanisms underlying reading disabilities in both atypical readers and children with ADHD-I.

To conclude our study, we conducted linear regression to extract the final adjusted models that best explain the number of words read correctly in 60'' and 180'', both measures of reading speed. To accomplish this, we initially recoded the diagnostic variable into two binary variables, "DiagRecDis" for children with dyslexia and "DiagRecDa" for children with ADHD-I. The analysis of these data led us to conclude that the lexical properties that most influence the number of words read correctly in 60'' and 180'' are, respectively, medium and short-length medium-frequency words, specifically total fixation time (TFT) in the former case and the second reading time (SPRT) in the latter. Our data suggests that the number of words read correctly in the first minute depends on the neuropsycholinguistic profile (dyslexia *versus* ADHD-I), visual attention, immediate visual memory, lexical access capacity, and total fixation time on medium length medium-frequency words. The fact that the total fixation time of medium length medium-frequency words influences reading fluency corroborates the finding that the early stages of the reading task activate more attentional and decoding mechanisms using phonological representations. Similarly, the number of words read correctly in 180'' is also influenced by the neuropsycholinguistic profiles associated with developmental dyslexia and ADHD-I and, by second reading pass (SPRT) on short medium-frequency words. It was possible to conclude that, as reading time increases, there is less activation of attentional mechanisms associated with reading and higher times to integrate the word in syntactic and semantic contexts, both reflecting late processing effects. The inclusion of the independent variable "DiagRecDa" in all three models confirms that children with ADHD-I, like their dyslexic peers, experience difficulties in reading fluency and accuracy. Their better performance in these tasks stems from the involvement of distinct cognitive mechanisms compared to dyslexia. This data can help find a biomarker based on eye movements, which according to Panagiotidi et al. (59) could be a very effective way of testing for ADHD traits.

The findings of this study can contribute to the development of targeted interventions for children with dyslexia and ADHD-I in several ways: 1) personalized interventions: understanding the distinct cognitive mechanisms underlying reading difficulties in these populations allows for the development of personalized interventions; 2) early identification and intervention: this research may help in the early identification of individuals at risk of dyslexia or ADHD-I; 3) improved educational strategies: educational professionals can use these findings to adapt teaching strategies; 4) enhanced resource allocation: schools and institutions can allocate resources more effectively; 5) research-based practices: this study adds to the knowledge base of evidence-based practices; 6) cross-collaboration: this research serves as a bridge connecting studies conducted in Portugal with international research, particularly in cases where different types of orthographies with varying levels of opacity pose unique research challenges and 7) policy implications: these findings may have implications for policy development and resource allocation. In summary, these findings provide valuable insights into the reading difficulties of children with dyslexia and ADHD-I and may contribute to the development of targeted interventions for these populations. It is important to consider these cognitive profiles in understanding the unique challenges faced by individuals with ADHD-I and atypical readers and tailoring appropriate interventions or support.

Finally, it is important to address some limitations of this study, particularly the sample size and issues related to differential diagnosis. Additionally, we will provide indications for future research directions. Concerning the former limitation, while we believe that the statistical methods we employed were robust enough to mitigate this constraint, we plan to increase the sample size in a future edition of this work to enhance the generalizability of our conclusions to the target populations studied. As for the latter limitation, we acknowledge that we are not immune to the challenges faced in other studies regarding sample selection and the distribution of participants across clinical groups. As mentioned in other sections of this work, there is a high comorbidity between both disorders. We believe that many of the doubts and incorrect conclusions that have arisen in other research regarding the sharing of the same cognitive deficits by both clinical conditions are due to diagnostic errors in the participant selection stage. In our study, we believe that the identification of distinct neuro-psycholinguistic traits in dyslexics and children with ADHD-I helped mitigate the effects of comorbidity. As for the next steps to take, we plan to incorporate data from the detection of microsaccades (small involuntary eye movements that occur once or twice per second during attempts at visual fixation) into the models obtained through linear regression, as these are relevant to visual perception, cognition, and oculomotor control and exhibit distinct characteristics in visual and oculomotor pathologies.

### Ethics and Conflict of Interest

The author(s) declare(s) that the contents of the article are in agreement with the ethics described in http://biblio.unibe.ch/portale/elibrary/BOP/jemr/ethics.html and that there is no conflict of interest regarding the publication of this paper.

### Acknowledgements

This research was supported in part by grant PEst-OE/LIN/UI0214/2013 from Fundação Ciência Tecnologia (FCT).
